# Computational Simulation Tools to Support the Tissue Paper Furnish Management: Case Studies for the Optimization of Micro/Nano Cellulose Fibers and Polymer-Based Additives

**DOI:** 10.3390/polym13223982

**Published:** 2021-11-18

**Authors:** Flávia P. Morais, Ana M. M. S. Carta, Maria E. Amaral, Joana M. R. Curto

**Affiliations:** 1 Fiber Materials and Environmental Technologies Research Unit (FibEnTech-UBI), Universidade da Beira Interior, R. Marquês d’Ávila e Bolama, 6201-001 Covilhã, Portugal; mecca@ubi.pt; 2 Forest and Paper Research Institute (RAIZ), R. José Estevão, Eixo, 3800-783 Aveiro, Portugal; ana.carta@thenavigatorcompany.com; 3 Chemical Process Engineering and Forest Products Research Centre (CIEPQPF), University of Coimbra, R. Sílvio Lima, Polo II, 3004-531 Coimbra, Portugal

**Keywords:** absorption, cellulose fibers, computational simulation, furnish optimization, modeling, polymeric additives, softness, strength, tissue papers

## Abstract

Tissue paper production frequently combines two main types of raw materials: cellulose fibers from renewable sources and polymer-based additives. The development of premium products with improved properties and functionalities depends on the optimization of both. This work focused on the combination of innovative experimental and computational strategies to optimize furnish. The main goal was to improve the functional properties of the most suitable raw materials for tissue materials and develop new differentiating products with innovative features. The experimental plan included as inputs different fiber mixtures, micro/nano fibrillated cellulose, and biopolymer additives, and enzymatic and mechanical process operations. We present an innovative tissue paper simulator, the *SimTissue*, that we have developed, to establish the correlations between the tissue paper process inputs and the end-use paper properties. Case studies with industrial interest are presented in which the tissue simulator was used to design tissue paper materials with different fiber mixtures, fiber modification treatments, micro/nano fibrillated cellulose, and biopolymer formulations, and to estimate tissue softness, strength, and absorption properties. The *SimTissue* was able to predict and optimize a broader range of formulations containing micro/nanocellulose fibers, biopolymer additives, and treated-fiber mixtures, saving laboratory and industrial resources.

## 1. Introduction

Furnish optimization is a multiple-input multiple-output (MIMO) challenge, mainly due to the different fiber and structure properties and the influence of several process operations. The development of multi-structured materials, such as tissue paper products, will benefit from the modeling of raw material characteristics and their functional properties. The use of computational tools constitutes an innovative strategy to enhance this type of polymeric materials, through three-dimensional (3D) modeling of fibers and structures.

Tissue papers, such as toilet paper, paper towels, napkins, and facial tissue masks, are cellulose fibrous materials designed for personal hygiene and utility purposes. Significative growth in the global tissue sector is projected for the next decade, concentrated in the industrialized parts of the world, and a demand for premium products [1]. The properties required for the tissue materials, such as softness, strength, and absorption, and the production process steps, such as creping and converting, distinguish them from other cellulose-based materials [2,3,4,5,6]. In general, the creping process consists of the formation of uniform roughness on the paper, enhancing the properties of bulk, softness, absorption, and elasticity [3]. The converting process includes diverse operations performed on base tissue paper sheets to develop a multi-layer finished product, ready to be placed on the market [3].

The properties of each tissue paper depend on the pulps used for its production, which are influenced by the type of pulp cooking and the applied bleaching sequence, as well as the fibrous raw materials, such as hardwoods like eucalyptus, or softwoods like Norway spruce or pine [7,8,9,10,11]. In the manufacturing of these products, a high percentage of hardwood pulps is employed, which provide structural and softness properties to the materials, with the incorporation of softwood pulps, which provide paper strength and ensure the paper machine runnability [3,9]. Eucalyptus fibers present an optimization potential to improve their mechanical properties while maintaining existing benefits. Maximizing the incorporation of these raw materials and the total or partial replacement of softwood pulps present challenges on the final creping and converting operations, specific to tissue paper materials. Obtaining tissue products with the best combination of fibers, and maximizing eucalyptus fibers, requires knowledge of the different pulps available on the market suitable for this type of materials and the consequences on the final end-use properties [9,10]. The management and optimization of tissue furnish play a key role to maximize eucalyptus fibers without significant losses in strength and runnability [3,11]. To enable the great potential of hardwood pulps in these materials, it is necessary to identify the fiber properties, their relationship with the processes, and the final end-use properties [9]. Therefore, the raw material selection, pulp cooking type, and bleaching sequences, fiber modification treatments such as refining and enzymatic processes, and the nature of additives added to the pulp suspension are crucial steps and factors that can affect the tissue key properties and consequently the final product [12]. These factors allow the modification of eucalyptus pulps, minimizing the influence on the strength and stiffness properties that the structure needs for the process operations.

The methods currently used for fiber modification include mechanical refining treatments, enzymatic biorefining treatments, and additive incorporation. These modification process steps are optimized to obtain improved functional properties with the maximization of fibers suitable for tissue papers. Refining is a fiber fibrillation process used to develop wet tensile strength, improve sheet formation, and modify fiber drainability. Consequently, this process decreases the softness and absorption properties by increasing the sheet density and elasticity modulus [13,14]. However, refining requires a substantial amount of energy. The use of enzymes reduces the refining time and the applied energy to achieve the desired properties. This green sustainable integrated process increases the paper softness properties, with better tensile strength, which facilitates an increase in machine speed, with a refining energy reduction [15,16]. The additives incorporation is also a strategy to optimize the performance of the enzyme in the tissue field, because they improve the enzymatic activity rate, allowing for a lower end-use enzyme application rate. The polymeric additives improve the tissue softness and/ or absorption properties, maintains or increases the paper strength properties, which can provide energy and cost savings [17,18,19,20]. However, balancing softness and strength properties in tissue paper production is not easy, as actions required to maximize softness are often in conflict with the ones needed to improve strength [3,7,9,10]. In our previous publications, we have proven that the incorporation of polymeric additives such as micro/nanofibrillated cellulose (MFC/NFC) and a commercial biopolymer into tissue structures enhance the maximization of eucalyptus fibers, optimizing the process and associated manufacturing costs, in order to produce high-quality products [17,18].

Consequently, a detailed level of knowledge is required when manufacturing these products to fulfill the high-quality requirements. It is also important to investigate various modification processes that allow the same or improved final end-use properties by maximizing the eucalyptus fibers incorporation and saving laboratory and industrial resources. An experimental design that included the production of laboratory-made structures to simulate industrial-made structures with enzymatically and mechanically treated fibers, additives incorporation, and even the physical changes of creping and converting operations, is essential to improve the tissue industrial process [21,22,23]. These approaches allow the introduction on the market of innovative and value-added formulations, such as antimicrobial tissue papers, individual protection tissue masks, and cosmetic facial tissue masks.

Predicting the tissue material properties from experimental data of pulps used in their production could be an important and interesting milestone to the tissue paper industry. The integration of all the important variables for premium tissue materials development with furnish management and optimization can be accomplished using computational tools, modeling, validation, and optimization methods, in order to design new tissue products with innovative features and added value. This approach allowed us to develop mathematical models, based on datasets, with predictive capacity for softness, strength, and absorption. Establishing relationships between fiber properties, process modification steps, and functional tissue properties, as well as the models that describe them, constitute a multifactorial challenge. Over the years, material modeling has become an important tool in the development and design of materials with better performance [24,25,26,27,28,29,30,31,32,33,34,35,36,37]. Our strategy also consists of 3D modeling of structured materials, such as tissue papers, considering the structural hierarchy at the fiber and structure level. In our previous work, an innovative 3D voxel approach was used to model the fibers and structures made by them, and consequently, to predict and optimize the properties of various types of tissue materials [26,27,35]. The computational tools allow us to significantly reduce the experimental work in the new materials development, to have a better synergy between the experimental tests design and the simulation of materials specific properties [35]. Despite its importance and the advances achieved in the field of fibrous materials science, engineering, and modeling, to the best of our knowledge, an approach to simulate and optimize the relationships between the key parameters and the functional properties of tissue materials is not presented in the literature. The advanced computational tools development with predictive capacity allows determining which are the most promising pulps for a specified tissue material and which process operations allow obtaining a tissue material with optimized properties. Therefore, we propose a materials design strategy using an innovative computational tool that is applied to develop cellulosic fiber-based materials, including polymeric additives at micro and nanoscale.

The main goal of this work was to present and compare different tissue furnish formulations, using a computational simulation tool with predictive capacity developed by us, named *SimTissue*, valuable to design new tissue multi-structured paper materials with innovative functionalities at the micro and nanoscale and polymer-based additives. This simulator establishes relationships between the key fiber properties, the fiber modifications process steps, and the tissue paper structural and functional properties for furnish management and optimization.

## 2. Materials and Methods

### 2.1. Pulp Samples

In this study, different pulps were selected, obtained from hardwood and softwood species and industrial processes relevant to the management of tissue papers. The pulps were disintegrated according to the ISO 5263/1. The fibers’ morphological properties were determined automatically by image analysis of a diluted suspension (20 mg/L and 30 mg/L for hardwood and softwood samples, respectively) in a flow chamber in a MorFi Fiber Analyzer (TECHPAP, Grenoble, France). After their characterization, the hardwood and softwood pulps were mixed in ratios of 99:1, 95:5, 90:10, and 75:25, where this last ratio is taken as an industrial reference.

### 2.2. Enzymatic Treatments

Two bleached eucalyptus pulps, with different cooking processes, kraft and sulfite, were treated separately, using enzyme dosages of 10 and 100 g per ton of pulp, for 30 and 60 min. The assays were carried out at a consistency of 4%, pH 7, and 40 °C, with continuous mechanical agitation. A minimum amount of sodium hypochlorite was added to the pulp suspension to stop the enzyme reaction. After these enzymatic treatments, the pulps were mixed in a ratio of 80:20. A more detailed description of this study can be found in [16].

### 2.3. Mechanical Treatments

A bleached eucalyptus kraft pulp was subjected to mechanical refining treatments using a PFI mill at 500, 1000, 2000, and 3000 revolutions, and two refining intensities of 3.33 N/mm, according to the ISO 5264-2 standard, and of 1.67 N/mm, which corresponds to half of the normalized value. This study was carried out to simulate processing conditions with different refining energies. The *SimTissue* was used in these studies to reduce energy consumption in the tissue process.

### 2.4. Combination of Enzymatic and Mechanical Treatments

To study the effect of the combination of both treatments (Section 2.2 and Section 2.3), different enzymatic treatments and a slight mechanical treatment were applied to the kraft pulp used in Section 2.2.

In these assays, enzyme dosages of 0.5 and 1 kg per ton of pulp were used, for 2 h, with the same process conditions noted in the enzymatic treatment method section. Subsequently, this pulp was beaten in a PFI mill at 500 revolutions, under a refining intensity of 3.33 N/mm. A pre-refining process followed by enzymatic treatment was also carried out under the same conditions.

### 2.5. Additives Incorporation

Different furnish mixtures were performed to understand the effect of polymer-based additives in the tissue properties compared to the reinforcing fibers frequently used in these types of products. The micro/nanofibrillated cellulose (MFC/NFC) was incorporated in a never-dry (slush) bleached eucalyptus kraft pulp, according to loads of 1%, 5%, and 10%. A commercial biopolymer additive was also incorporated in the same fiber pulp slurry, at the dosage of 2%. A more detailed description of this study can be found in [17,18]. This study was designed and predicted using the *SimTissue* to evaluate the potential for reducing or replacing softwood reinforcement fibers with these polymeric additives.

### 2.6. Handsheets Preparation and Testing

The tissue structures were produced in a batch laboratory sheet former according to an adaptation of ISO 5269-1. The adjustments consisted of the production of structures with a basis weight of 20 g/m^2^ instead of 60 g/m^2^ and suppression of the pressing operation as described in the respective standard. This experimental methodology allowed us to obtain sheet structures similar to tissue papers. The tissue structures were prepared according to the different process steps described above. Additionally, all samples were conditioned at 23 ± 1 °C, with a relative humidity of 50 ± 2%, according to ISO 187.

For all assays, the tissue properties for handsheets were measured in terms of structural and functional properties. The basis weight (ISO 12625-6) was obtained by the quotient between the average mass of each structure and the respective area (0.02138 m^2^). The thickness (ISO 12625-3) was obtained using a Frank-PTI micrometer. The bulk (ISO 12625-3) was obtained by the quotient between the structure thickness and the basis weight. The apparent porosity was calculated according to the method described by Morais et al. [18]. The softness, namely the handfeel (HF) parameter, was evaluated using a Tissue Softness Analyzer (TSA, Emtec equipment). The tensile index was assessed according to ISO 12625-4. The water absorption capacity was determined with the immersion absorption method, according to an adaptation of ISO 12625-8. The Klemm capillary rise method was evaluated according to an adaptation of ISO 8787. A more detailed description of these adaptations can be found in our referenced and cited publications [9,10,16,17,18].

### 2.7. Computational Simulator

A tissue computational simulator developed from scratch, the *SimTissue* (FibEnTech-UBI members, Covilhã, Portugal) was used to compare and predict different tissue furnish formulations, with different enzymatic and mechanical treatments, and additives incorporation from those studied experimentally. This simulator can establish the correlations between the tissue paper process inputs and the end-use paper properties. The ability to predict different scenarios was facilitated using advanced computational tools, including multiple and robust linear regressions, interpolation and extrapolation methods, and a 3D simulator of fibrous materials named *voxelfiber* [26,27]. The *voxelfiber* allows the material modeling and simulation as planar random networks, through the fiber dimensions and properties [35], and it can be accessed in https://github.com/eduardotrincaoconceicao/voxelfiber (accessed on 1 September 2021). Computational studies were carried out using MATLAB^®^ (R 2020a, 9.8.0.1323502, MathWorks, Natick, MA, USA). All variables were normalized to present the same scale range.

Statistical analysis was performed using cluster analysis with IBM SPSS Statistics 25 (Armonk, NY, USA). The hierarchical cluster analysis was used, with Ward’s method and square Euclidean distance measure. In the proximity matrix, a range of solutions of 2 to 3 clusters was used.

## 3. Results and Discussion

### 3.1. Pulp Samples Characterization

**Figure 1:**
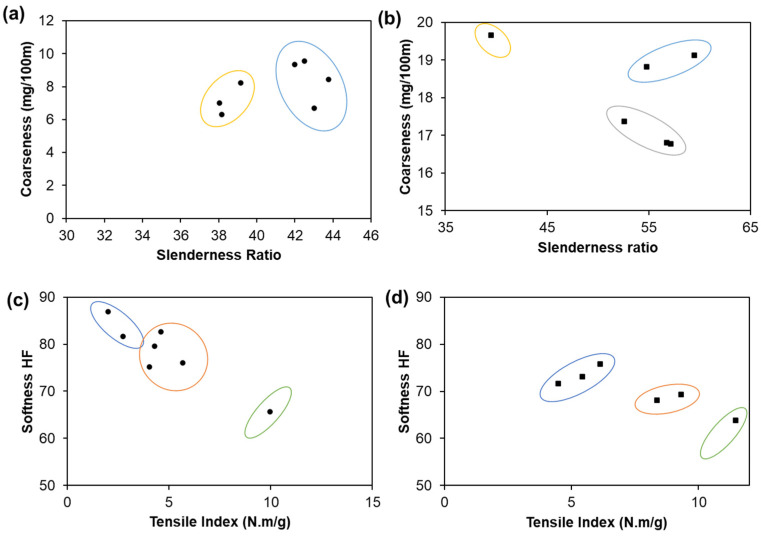
Coarseness as a function of slenderness ratio of hardwood (**a**) and softwood (**b**) fiber pulps, as well as softness HF as a function of tensile index properties of hardwood (**c**) and softwood (**d**) fiber pulps.

An experimental set of different hardwood and softwood fiber pulps suitable for tissue materials production was characterized in terms of fibers morphology and suspensions drainability accessed by the Schöpper-Riegler method, °SR [9,10]. Hardwood fibrous suspensions present ranges between 18 and 25 °SR and softwood suspensions between 12 and 15 °SR. Figure 1 shows the relationship between the slenderness ratio and coarseness, as well as between the TSA-softness and tensile index properties of the hardwood and softwood fiber samples. The low slenderness ratio values of hardwood fibers (38–44) represent fibers with low flexibility and low chance of forming well-bonded structures, resulting in high softness properties, compared to the high slenderness ratio values of softwood fibers (39–59), resulting in improved strength properties. The hardwood fibers studied can be separated into two clusters of slenderness ratio vs. coarseness, whereas the softwood fibers are classified into three distinct clusters (Figure 1a,b). The organization of these clusters corresponds to a similar behavior between pulp fibers, indicating the direct or inverse relationship between properties. In Figure 1a, for example, the hardwood pulps grouped in the yellow cluster presented a positive correlation. The statistics results indicated that the coarseness increased by 1.4 units per unit of the increased slenderness ratio, with a R-squared (coefficient of determination—R^2^) of 0.8. In contrast, in the blue cluster, these pulps presented a negative correlation (Figure 1a). The statistics results indicated that the coarseness increased by 0.8 units per unit of the decreased slenderness ratio, with a R^2^ of 0.3. All this cluster analysis was performed to set similar cases into groups, being distinct from each other. The data were based on our previous work in the analysis of a representative pulp group to produce tissue materials, namely seven hardwood pulps and six softwood pulps. For this reason, there are few points in each cluster. However, these data were adequate to feed our computational simulator, *SimTissue*. These are samples without any enzymatic and/or mechanical treatments or additives incorporation, which does not occur in the industry. It is important to emphasize the importance of these data to select the most suitable raw material to be optimized in the process modification steps.

Hardwood fibers with a slenderness ratio between 38 and 39 and coarseness between 6.31 and 8.22 mg/100 m represent the highest softness values (81.7–86.9 HF). In contrast, hardwood fibers with a slenderness ratio between 42 and 43 and coarseness between 6.71 and 9.56 mg/100 m represent, on average, the highest values of the tensile index (4.03–9.96 Nm/g). Softwood fibers with a slenderness ratio between 39 and 59 and coarseness between 18.83 and 19.66 mg/100 m present high softness (71.3–75.8 HF) and low tensile index (4.49–6.12 Nm/g). Additionally, softwood fibers with a slenderness ratio between 52 and 57 and coarseness between 16.77 and 17.37 mg/100 m present high tensile index values (8.36–11.45 Nm/g) and consequently low softness values (63.8–69.3 HF).

Regarding the tensile index vs. TSA-softness properties, the samples were classified into three clusters (Figure 1c,d). The results indicate that two hardwood pulps with low tensile index values and high softness values belong to cluster 1 (blue), the four pulps with intermediate values for both properties belong to cluster 2 (red), and one pulp stands out from the rest for being isolated in cluster 3 (green), presenting the highest values of the tensile index. The same analogy was made for softwood pulps, and one pulp also stands out from the rest due to the higher values of the tensile index. Overall, softness and strength properties are inversely related. Hardwood eucalyptus slush pulp was also used for the studies. The particularity of this sample is to present high values of softness (82.7 ± 4.0 HF) and intermediate values of the tensile index (4.60 ± 0.61 Nm/g). Although this classification is not the same in both analyses presented in Figure 1, there is a relationship between the morphological properties of length/width ratio and coarseness and the tissue functional properties of softness and tensile index.

Water absorbency properties are essential for the good performance of tissue papers and depend on the structural tissue properties, namely the thickness, bulk, and porosity. The characterization of tissue apparent porosity is crucial, as the ability of the liquids to migrate through the tissue paper depends on the pore sizes [38,39]. The complexity of these porous materials comes from the fact that they do not have a regular pore distribution, but rather a pore dimension that varies in a wide range and the tortuous paths that the pores provide for the flow of the liquid [40]. The molecules need to travel a path higher than a straight line between the original source and its active local [41]. Therefore, 3D structural porous materials structure knowledge is important for the prediction of water absorption properties [25].

**Figure 2:**
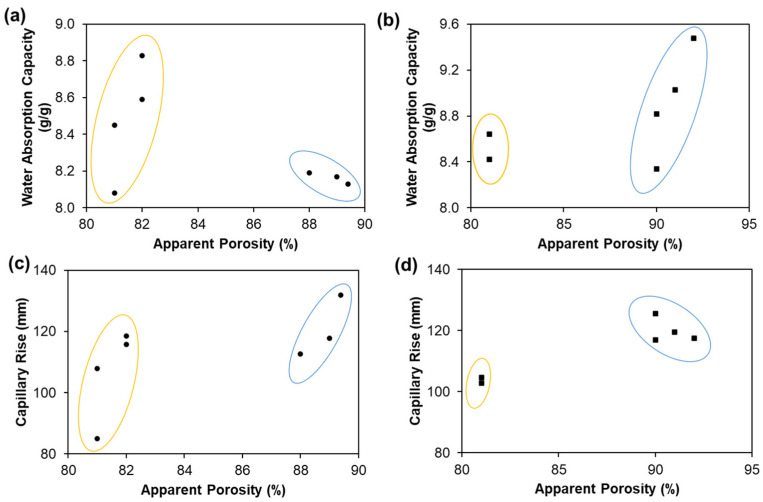
Water absorption capacity as function of apparent porosity of hardwood (**a**) and softwood (**b**) fiber pulps, as well as capillary rise as function of apparent porosity of hardwood (**c**) and softwood (**d**) fiber pulps.

Figure 2 shows the water absorption capacity and capillary rise–Klemm method properties as a function of the apparent porosity properties for hardwood and softwood handsheets. The clusters involved in these relationships are identified as well as their positive and negative correlation. For example, in a yellow cluster of hardwood pulps from Figure 2a, the statistics results indicate that the water absorption capacity increased by 0.4 units per unit of the increased apparent porosity, with a R^2^ of 0.7. In contrast, in a blue cluster, the statistics results indicated that the water absorption capacity increased by 0.04 units per unit of the decreased apparent porosity, with a R^2^ of 0.8 (Figure 2a). The same analogy was made for softwood pulps and capillary rise properties. According to Figure 2a,c, the results indicated that hardwood fibers with porosities between 81 and 82% present water absorption capacity between 8.08 and 8.83 g/g and capillary rise between 85 and 119 mm. Hardwood fibers with higher porosity (88–89%) presented water absorption capacity between 8.13 and 8.19 g/g and capillary rise between 113 and 132 mm. According to Figure 2b,d, the results indicated that the softwood fibers with 81% porosity present water absorption capacity between 8.42 and 8.64 g/g and capillary rise between 103 and 105 mm. Softwood fibers with higher porosity (90–92%) present water absorption capacity between 8.34 and 9.48 g/g and capillary rise between 117 and 126 mm.

Considering the previous characterization, different pulp samples were selected for the studies of fiber modification process operations and polymer-based additives incorporation. For the enzymatic modification studies, hardwood fiber samples with extreme values of softness (86.9 ± 3.1 HF) and tensile index (9.96 ± 0.30 Nm/g) properties were selected. For the refining studies, we selected the hardwood pulp with a balance between the properties of softness (81.7 ± 3.3 HF), tensile index (2.73 ± 0.42 Nm/g), and water absorption capacity (8.59 ± 0.22 g/g). For the enzymatic and mechanical treatment combination studies, we selected the hardwood pulp with the highest strength properties (9.96 ± 0.30 Nm/g). Additives were incorporated with the eucalyptus slush pulp. Finally, fiber mixtures were carried out using the latter pulp sample and the softwood pulp with the extreme values of the tensile index (11.45 ± 0.99 Nm/g).

### 3.2. Predictive Capacity for Furnish Optimization

#### 3.2.1. Fiber Mixtures Ratio

**Figure 3:**
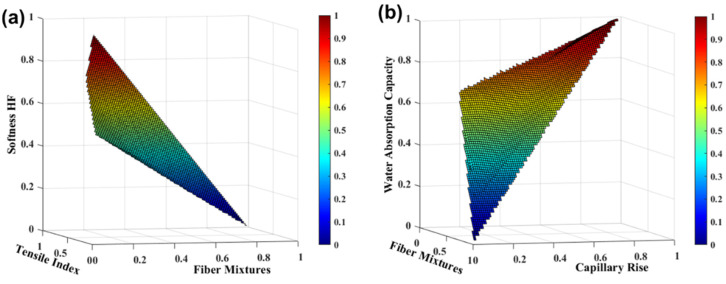
TSA-Softness HF and tensile index properties (**a**) and water absorption capacity and capillary rise (**b**) with eucalyptus fiber and softwood fiber mixtures. The variables were normalized.

A blend of hardwood pulps with a percentage of softwood pulps is used to produce these materials. The softwood fibers ensure the fibrous structure strength and runnability to the tissue paper machine; however, these fibers present a high cost in tissue production. Therefore, it is essential to find effective strategies for removing or replacing these fibers in the production of these products. In our work, we investigated mixtures of eucalyptus fiber and softwood reinforcement fiber in ratios of 100:0, 99:1, 95:5, 90:10, and 75:25. The experimental results indicated that the strength properties were increased from 93 to 303% and the properties of softness, water absorption capacity, and capillary rise were decreased from 28 to 8%, 7 to 1%, and 7 to 2%, respectively [17]. The *SimTissue* allows us to predict a wide range of scenario variability by quantifying the influence of incorporating other percentages of reinforcement fibers that have not been studied experimentally. The ideal ranges for tissue properties were obtained simultaneously through computational simulation to identify different combinations of fiber percentages. All these computational combinations allow covering a much larger results area, which would not be possible using only experimental results, with the time and resources they require [17]. Figure 3 presents the functional properties of tissue products as a function of fiber mixtures, obtained with the *SimTissue* prediction. For the studies, we considered the softwood maximum incorporation of 30%, as tissue papers are produced in this range. Higher softness values correspond to lower tensile index values. This trend varies with the fiber mixture, where the normalized variable 0 corresponds to a formulation with 100% eucalyptus pulp, and the normalized variable 1 corresponds to a formulation with 70% eucalyptus pulp and 30% softwood fibers, the reasonable maximum incorporation to produce tissue papers.

The computational results indicated that the incorporation of 30% reinforcement fibers in a tissue structure increases the tensile index properties by 330% and decreases the properties of softness by 30%, water absorption capacity by 9%, and capillary rise by 7%. Through our *SimTissue*, we found that the balance between the properties of softness (65.0–69.3 HF), strength (14.5–15.9 Nm/g of the tensile index), and absorption (7.8–8.0 g/g of water absorption capacity and 38.3–39.5 mm of capillary rise) can be found with the incorporation of 5 to 15% of reinforcement fibers in tissue papers. This suggests the possibility of reducing the incorporation of softwood fibers in tissue structures, which translates into financial advantages. The results presented in this section describe structures without process modification steps. Therefore, to balance the absence of softwood pulps, it is also important to select strategies to modify the fibers combining the enzymatic and mechanical fiber modification treatments, as well as the incorporation of polymeric additives in the suspensions.

#### 3.2.2. Enzymatic Treatment

**Figure 4:**
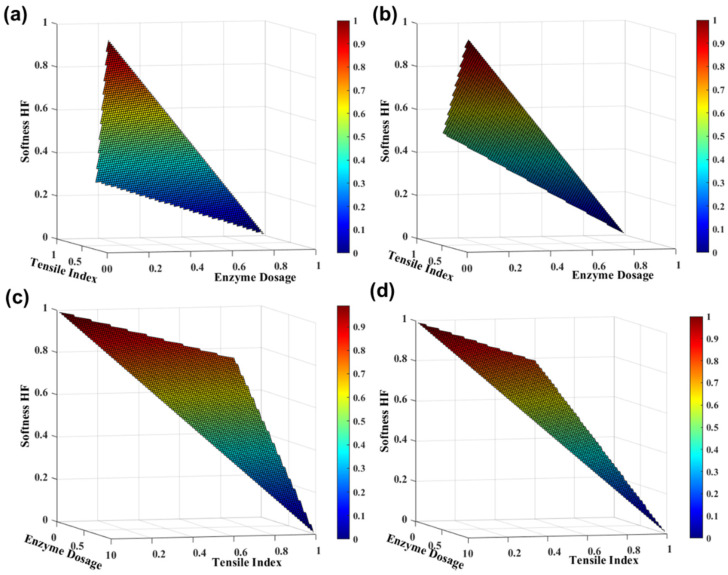
TSA softness HF and tensile index properties with enzyme dosages in eucalyptus kraft pulp (**a**,**b**) and eucalyptus sulfite pulp (**c**,**d**) for 30 min (**a**,**c**) and 60 min (**b**,**d**). The variables were normalized.

New enzymatic technologies reduce refining energy and consequently production costs, and could also optimize the tissue functional properties, that one may develop new products with high added value. In our previous work, an approach to optimize two eucalyptus fiber pulps was used through different enzymatic treatments. The sulfite and kraft pulps were treated to obtain optimized softness and strength properties, respectively, to produce tissue structures with 100% eucalyptus fibers [16]. Figure 4a,b presents the TSA-softness HF and strength properties as a function of enzyme dosage in eucalyptus kraft pulp at 30 and 60 min, obtained with the *SimTissue* forecast. The simulations predicted the functional properties of different formulations with variation in the two process variables: reaction time and enzyme dosage. We considered a maximum enzyme dosage of 100 g/ton. Regarding the enzymatic treatment in the kraft pulp, the results indicated that in a time reaction of 30 and 60 min, the softness properties are decreased by 20% and 29%, respectively, and the strength properties are increased by 2%, with increasing enzyme dosage. Through our *SimTissue*, we found that the balance between the properties of softness (53.0 ± 4.0 HF) and strength (18.5 ± 2.0 Nm/g) can be found with an enzyme dosage of 50 g/ton for 30 min or 20 g/ton for 60 min. In these simulation studies, similar softness and strength properties are obtained with a lower reaction time (30 min) and higher enzyme dosage (50 g/ton), or also with twice the reaction time (60 min) and an enzyme dosage 2.5 times less (20 g/ton). Additionally, these results indicated that these final properties are in the same variation range as the formulation typically used in the tissue industry, namely 75% eucalyptus pulps + 25% softwood pulps (softness: 59.9 ± 2.3 HF and tensile index: 18.5 ± 1.1 Nm/g).

This analysis is also performed for other suitable pulps in tissue paper production. Regarding the enzymatic treatment in the sulfite pulp, the results indicated that in a timed reaction of 30 and 60 min, the softness properties are decreased by 0.1%, and the strength properties are increased by 2% and 4%, respectively, with increasing enzyme dosage (Figure 4c,d). *SimTissue*’s predictive ability indicated that the balance between softness properties (82.0 ± 5.0 HF) and strength properties (7.0 ± 2.1 Nm/g) can be achieved with an enzyme dosage of 100 g/ton for 30 min or 30 g/ton for 60 min.

With this approach, strategies were found to decrease the enzyme dosage in eucalyptus fiber modification treatments, increasing the reaction time of the enzyme. These results suggest a decrease in the economic value of the formulations associated with the enzyme costs.

#### 3.2.3. Mechanical Treatment

**Figure 5:**
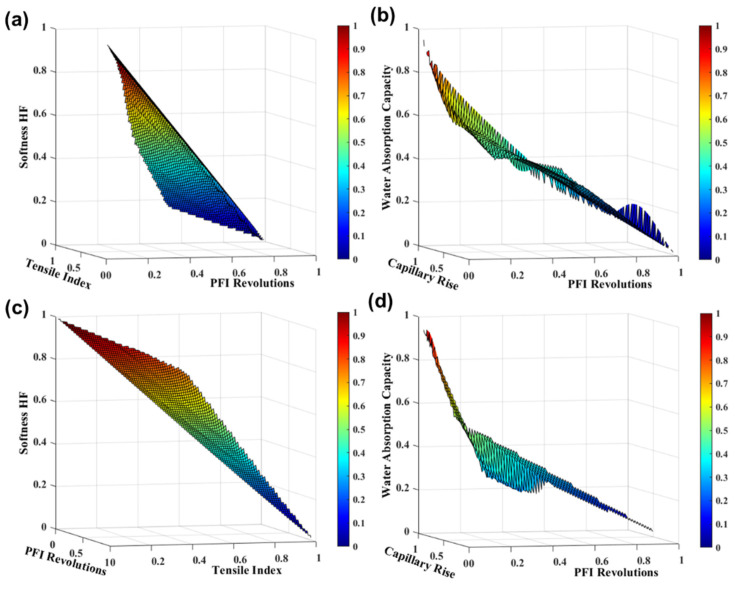
TSA-softness HF and tensile index properties (**a**,**c**) and water absorption capacity and capillary rise (**b**,**d**) with PFI revolutions at 3.33 N/mm (**a**,**b**) and 1.67 N/mm (**c**,**d**). The variables were normalized.

The mechanical treatment of refining is also one of the fundamental steps in tissue paper production. The drainage characteristics between 25 and 32 °SR to produce high-quality tissue sheets are desirable [3]. As all these characteristics are affected during refining processes, mechanical treatment optimization must be considered. Our strategy was to investigate the influence of the refining intensity on the tissue functional properties of eucalyptus pulp to save energy resources from refining. Figure 5 present the evolution of the final end-use tissue properties as a function of the PFI revolutions at 3.33 N/mm and 1.67 N/mm, obtained by *SimTissue*. These results indicated that similar tissue properties are achieved in 1000 PFI revolutions at 3.33 N/mm and 600 PFI revolutions at 1.67 N/mm. In these conditions, the strength properties are increased by 140%, and softness HF, water absorption capacity, and capillary rise are decreased by 60%, 15%, and 20%, respectively. Simulation studies were used to optimize the mechanical treatments, with different refining energies, obtaining similar tissue properties with higher or lesser refining times and intensities. A strategy of reducing the refining intensity can be implemented in the industry, saving energy costs and consumption in tissue products production. This computational tool allows predicting the final properties, according to the pulp input characteristics, and therefore requires reliable databases. Different scenarios of industrial interest can be accurately evaluated, without extensive experimental planning design, to optimize raw material management and associated formulation costs. The integration of all the variables of an industrial tissue process will dynamically model the evolution of the tissue process and study the effect of changes and disturbances over time in this industrial process for forecasting and decision-making. This allows assessing expenses and operational implications in each scenario simulated by *SimTissue*.

#### 3.2.4. Combination of Enzymatic and Mechanical Treatments

**Figure 6:**
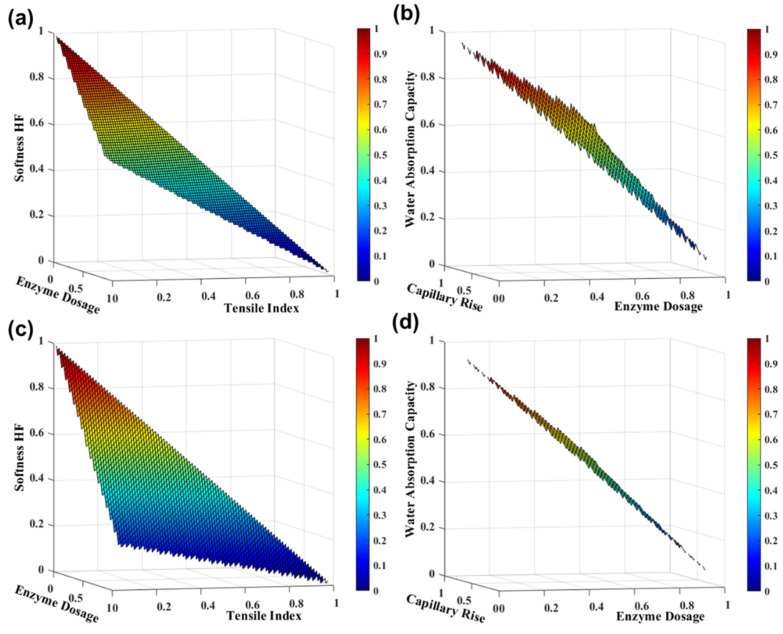
TSA-softness HF and tensile index properties (**a**,**c**) and water absorption capacity and capillary rise (**b**,**d**) with enzyme dosage in an enzymatic pretreatment process followed by 500 PFI revolutions at 3.33 N/mm (**a**,**b**) and pre-refining at 500 PFI revolutions at 3.33 N/mm, followed by enzymatic treatment (**c**,**d**). The variables were normalized.

Innovative methodologies and the optimization of the tissue paper production process are crucial strategies for managing tissue furnish. The application of an enzymatic pretreatment followed by a mechanical refining process is commonly used in the tissue industry. However, to the best of our knowledge, a mechanical pre-treatment followed by enzymatic treatment is not used industrially. The investigation and comparison of these two processes in eucalyptus pulp are essential to optimize the enzyme dosage in the production process as well as the savings of refining energy consumption and costs. Figure 6a,b presents the tissue functional properties as a function of enzyme dosage, in an enzymatic pretreatment process followed by 500 PFI revolutions at 3.33 N/mm, according to the *SimTissue* prediction. The results indicated that this process increased the strength properties by 6% and decreased the softness HF, water absorption capacity, and capillary rise by 15%, 8%, and 15%, respectively. The reverse process of pre-refining at 500 PFI revolutions at 3.33 N/mm, followed by enzymatic treatment increased the strength properties by 314% and decreased the softness HF, water absorption capacity, and capillary rise by 69%, 17%, and 41%, respectively (Figure 6c,d). These results indicate that a pre-refining followed by an enzymatic treatment is not advantageous to produce tissue papers with desired softness and absorption properties. However, this treatment is an innovative strategy to enhance strength properties. Therefore, a eucalyptus pulp with improved strength characteristics can be obtained through this fiber modification process. A planning design that combines this eucalyptus pulp with other pulps with good softness and absorption characteristics can be proposed. To this extent, formulations with 100% eucalyptus pulps and with optimized tissue properties can be used to produce high-quality products.

Additionally, to optimize the tissue furnish management, this tissue simulator predicted that the same tissue properties are achieved with an enzymatic pre-treatment using an enzyme dosage of 350 g/ton, followed by refining and with a pre-refining followed by enzymatic treatment with an enzyme dosage of 50 g/ton. This indicates that a potential savings of 85% in the enzyme dosage consumption can be achieved with the same energy consumption, obtaining properties of the softness of 60 ± 3 HF, tensile index of 8 ± 2 Nm/g, the water absorption capacity of 8 ± 1 g/g, and capillary rise of 95 ± 3 mm, which can be viably implemented in the tissue industry.

#### 3.2.5. Additives Incorporation

**Figure 7:**
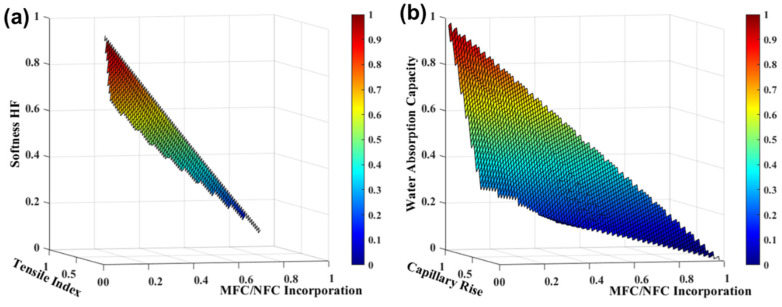
TSA-softness HF softness and tensile index properties (**a**) and water absorption capacity and capillary rise (**b**) with MFC/NFC incorporation in a eucalyptus slush pulp. The variables were normalized.

There is also a great diversity of additives in tissue paper production used to improve the tissue final end-use properties. Currently, additives such as softeners, wet strength agents, dry strength agents, retention agents, among others, are used. However, the use of more promising additives, as is the case of micro/ nanofibrillated cellulose (MFC/NFC), is still not widely considered industrially for this type of daily use papers. Gonzalez et al. [42] proved that highly fibrillated cellulose fibers enhanced significant strength increases in tissue structures that are strategically used to reduce the fiber content in formulations, maintaining the minimum functional strength and softness required for adequate performance and consumer satisfaction. Therefore, to produce an innovative tissue product with 100% eucalyptus fibers, we investigated and quantified the influence of the MFC/NFC incorporation, as an additive, in a eucalyptus slush pulp [17]. Figure 7 presents the final tissue properties as a function of the MFC/NFC incorporation at different dosages, through the *SimTissue* prediction. For these studies, we considered maximum incorporation of 15%, as MFC/NFC only had an additive function. These results indicated that the MFC/NFC incorporation in tissue structures increased the strength properties by 238% and decreased the softness HF, water absorption capacity, and capillary rise by 16%, 12%, and 22%, respectively.

Through *SimTissue*, we found that the balance between the properties of softness (74–79 HF), strength (7–11 Nm/g of the tensile index), and absorption (7.3–7.4 g/g of water absorption capacity and 34–38 mm of capillary rise) can be found with the MFC/NFC incorporation of 1 to 8% in tissue papers [17]. This additive can also be used as a substitute for softwood reinforcement fibers in tissue paper production, as improved properties are achieved in a tissue structure with 100% eucalyptus fibers at the micro and nanoscale, compared to structures with fiber mixtures as previously reported (Figure 3).

**Figure 8:**
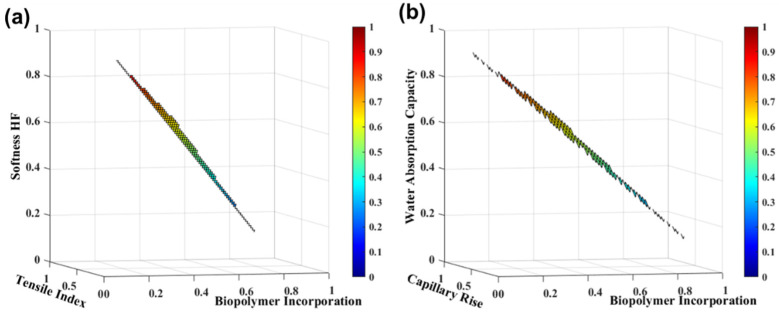
TSA-softness HF and tensile index properties (**a**) and water absorption capacity and capillary rise (**b**) with biopolymer incorporation in a eucalyptus slush pulp. The variables were normalized.

Dry strength agents, such as cationic starch, are used to increase the paper’s physical strength, increasing the polarity and affinity for anionic materials. However, this agent decreased the paper softness properties. Gonzalez et al. [42] also showed that the use of a cationic polymer in tissue formulations counteracts the adverse effects on drainability of high fibrillated fiber furnishes, ensuring that tissue paper machine runnability is not affected and that production rates remain at normal levels. Therefore, the use of a commercial biopolymer additive as a substitute for cationic starch that increases the strength properties while maintaining the softness properties by removing or replacing the softwood reinforcing fibers is also a good strategy to be considered industrially [18]. Figure 8 presents the tissue functional properties as a function of the biopolymer incorporation at different dosages in eucalyptus slush pulp, through the *SimTissue* forecast. For these studies, we considered maximum incorporation of 5%. The results indicated that the biopolymer incorporation increased the strength properties by 390% and decreased the TSA-softness HF, water absorption capacity, and capillary rise by 24%, 12%, and 42%, respectively.

The tissue paper simulator for furnish management optimization predicted that the balance between the softness (72 ± 2 HF), the strength (15 ± 2 Nm/g of the tensile index), and the absorption (8 ± 1 g/g of water absorption capacity and 97 ± 4 mm of capillary rise) can be found with the biopolymer incorporation of 3% in tissue papers, replacing the use of reinforcement fibers in these structures. The biopolymer incorporation of 4% still allows obtaining the same strength properties (18.7 ± 1.3 Nm/g) as the typical mixture used industrially (75 HW:25 SW) (18.5 ± 1.1 Nm/g), improving the softness (67.3 ± 1.9 HF vs. 59.9 ± 2.3 HF) and absorption (7.3 ± 0.6 g/g vs. 7.5 ± 0.4 g/g; 86 ± 4 vs. 38 ± 4 mm). The simulator’s predictive capacity allowed us to define several scenarios for the replacement or reduction of the softwood fiber using innovative polymer-based additives.

#### 3.2.6. Furnish Formulations to Develop Premium Tissue Materials

The development of premium tissue products requires the optimization of furnish formulations and, consequently, the functional tissue properties. The planning and management of innovative furnish formulations allow the choice of different fiber combinations, process steps, and additive incorporation using *SimTissue*’s predictive ability.

Table 1 presents different scenarios with an interest to produce various tissue papers, aiming at maximizing the trade-off between the softness, strength, and absorption properties.

This tissue simulator for furnish management and optimization allows the design of new innovative products, which can be achieved with the combination of two enzyme-treated eucalyptus kraft and sulfite pulps, in a ratio of 80:20 [16]. In formulation 1, both pulps were treated enzymatically with an enzyme dosage of 10 g/ton for 60 min. In formulation 2, only the eucalyptus kraft pulp was treated enzymatically with the same enzyme dosage for 30 min. Formulation 1 showed softness of 74 ± 5 HF and a tensile index of 13 ± 4 Nm/g. Compared to industrial reference mixtures (75% hardwood fibers + 25% softwood fibers), this formulation presents a 23% increase in softness HF and a 28% decrease in tensile index properties. Formulation 2 showed softness of 58 ± 3 HF and a tensile index of 18 ± 3 Nm/g. Compared to the same reference mixture, this formulation presents the same tensile index properties and a decrease of only 3% in the softness. The results can indicate that formulation 1 can be used to produce toilet papers or facial papers, where softness properties are essential, whereas formulation 2 can be used to produce towel papers or napkins, where strength properties are essential. These innovative formulations allow the production of tissue materials with 100% eucalyptus fibers, reducing the costs associated with softwood fibers.

Another approach may allow the design of new products with the combination of hardwood, softwood, and MFC/NFC cellulose fibers [17] (formulation 3 in Table 1). Formulation 3 showed softness of 71 ± 2 HF, tensile index of 14 ± 1 Nm/g, and water absorption capacity of 7 ± 0 g/g. Compared to industrial reference mixtures, this formulation presents an 18% increase in softness HF, a 23% decrease in tensile index properties, and a 2% decrease in water absorption capacity properties. The results indicate that formulation 3 can be used to produce a premium high-quality product where the compromise between the softness, strength, and absorption properties is optimized. This innovative formulation allows designing innovative tissue materials at the micro and nanoscale [17].

## 4. Conclusions

Computational simulation tools were applied to optimize tissue paper materials. The simulator allowed the integration of all the important variables for the tissue paper structures development. This innovative tool allowed the valorization of different cellulose fibers from renewable sources and polymer-based additives, resulting in possible financial improvements. *SimTissue*’s predictive capacity also allowed the development of innovative furnish formulations, containing micro/nanofibers and biopolymer additives, saving laboratory and industrial resources. In this study, different fiber mixing and modification process strategies were evaluated considering the desired softness, strength, and absorption properties, including maximizing the eucalyptus pulp incorporation, with the addition of MFC/NFC, biopolymer, different eucalyptus pulps, or softwood pulps, among others.

The results indicated that *SimTissue* evaluates the potential of raw materials for each type of tissue paper materials, considering the characteristics of fibers and structures made by them. The reduction of softwood fibers incorporation presented economic advantages, improving the functional properties. The simulations also predicted the properties of formulations with different enzyme reaction times and dosage, obtaining more economical scenarios associated with enzyme costs. The results also exemplified the possibility of using the simulator to reduce refining energy consumption, obtaining similar tissue properties with different refining times and intensities. With this computational approach, several scenarios for enzymatic and mechanical treatments with equivalent energy consumption were also foreseen. Finally, the *SimTissue* also allows us to predict scenarios in which the incorporation of additives such as MFC/NFC and biopolymers can be considered to replace softwood fibers to produce tissue products. The results indicated that formulations with 100% eucalyptus, when optimized with fiber modification treatments and polymer-based additives, can present improved properties over formulations with softwood fiber pulps.

In conclusion, the *SimTissue* is a useful tool not only to support industrial production in furnish management and process optimization but also to design innovative tissue materials, at the micro and nanoscale, establishing the correlations between tissue process inputs and the end-use properties.

## Figures and Tables

**Table 1:** Examples of case studies of different furnish scenarios to design new multi-structured innovative tissue paper materials.

Formulations	Hardwoods	Softwoods	Fiber Modification Process	Additives Incorporation
**1**	80% Eucalyptus kraft pulp + 20% Eucalyptus sulfite pulp	-	Both with enzymatic treatment of 10 g/ton, at 60 min	-
**2**	80% Eucalyptus kraft pulp + 20% Eucalyptus sulfite pulp	-	First with enzymatic treatment of 10 g/ton, at 30 min + Second without treatment	-
**3**	90% Eucalyptus kraft slush pulp	5% softwood kraft pulp	Beating process of softwood pulp achieving 25 °SR	5% MFC/NFC

## Data Availability

The raw/processed data required to reproduce the above findings cannot be shared at this time as the data also form part of an ongoing study.
